# Expression and Functional Characterization of Membrane-Integrated Mammalian Corticotropin Releasing Factor Receptors 1 and 2 in *Escherichia coli*

**DOI:** 10.1371/journal.pone.0084013

**Published:** 2014-01-17

**Authors:** Roberto Jappelli, Marilyn H. Perrin, Kathy A. Lewis, Joan M. Vaughan, Christos Tzitzilonis, Jean E. Rivier, Wylie W. Vale, Roland Riek

**Affiliations:** 1 Laboratory of Genetics, Salk Institute for Biological Studies, La Jolla, California, USA; 2 Laboratory of Neuronal Structure and Function, Salk Institute for Biological Studies, La Jolla, California, USA; 3 Laboratory of Physical Chemistry, ETH, Zürich, Switzerland; 4 Clayton Foundation Laboratories for Peptide Biology, Salk Institute for Biological Studies, La Jolla, California, USA; University of Saskatchewan, Canada

## Abstract

Corticotropin-Releasing Factor Receptors (CRFRs) are class B1 G-protein-coupled receptors, which bind peptides of the corticotropin releasing factor family and are key mediators in the stress response. In order to dissect the receptors' binding specificity and enable structural studies, full-length human CRFR1α and mouse CRFR2β as well as fragments lacking the N-terminal extracellular domain, were overproduced in *E. coli*. The characteristics of different CRFR2β -PhoA gene fusion products expressed in bacteria were found to be in agreement with the predicted ones in the hepta-helical membrane topology model. Recombinant histidine-tagged CRFR1α and CRFR2β expression levels and bacterial subcellular localization were evaluated by cell fractionation and Western blot analysis. Protein expression parameters were assessed, including the influence of *E. coli* bacterial hosts, culture media and the impact of either PelB or DsbA signal peptide. In general, the large majority of receptor proteins became inserted in the bacterial membrane. Across all experimental conditions significantly more CRFR2β product was obtained in comparison to CRFR1α. Following a detergent screen analysis, bacterial membranes containing CRFR1α and CRFR2β were best solubilized with the zwitterionic detergent FC-14. Binding of different peptide ligands to CRFR1α and CRFR2β membrane fractions were similar, in part, to the complex pharmacology observed in eukaryotic cells. We suggest that our *E. coli* expression system producing functional CRFRs will be useful for large-scale expression of these receptors for structural studies.

## Introduction

With approximately 800 different genes, G-protein-coupled receptors (GPCRs) constitute the largest family of human integral membrane proteins. By transducing a wide array of extracellular stimuli into intracellular signals, they participate in many fundamental biological processes [1]. In addition, because of their important role in pathological processes and cell membrane localization, they represent very prominent drug targets [2]. Structurally, GPCRs are characterized by an extracellular N-terminus, followed by seven transmembrane segments, which are connected by intracellular and extracellular loops, with the C-terminus located inside the cell. Based on their sequence similarity, they are divided in five classes. GPCR stimulants range from photons, odorants, and small molecules to large peptide and protein hormones. Upon ligand binding, GPCRs act as guanine nucleotide exchange factors for G proteins, which in turn modulate cellular enzymes or channels. The association of GPCRs with biological membranes makes their structural analysis especially challenging. This can be ascribed to difficulties in the expression, solubilization, purification, stabilization and crystallization of membrane proteins in general, and to the distinct flexibility of GPCR molecules. Despite some very remarkable recent progresses in the structural biology of GPCRs [3], [4], only relatively few full-length GPCR structures have been determined.

Corticotropin-releasing factor receptors (CRFRs) belong to the class B1 (secretin) family, which in mammals consists of 15 members and is distinguished by a relatively large N-terminal extracellular domain (ECD-1), which participates in the binding of endogenous polypeptide hormones [5]. Most of the sequence variation between the class B1 members resides in the ECD-1s. Based on structural and biochemical studies, class B1 ECD-1s adopt a distinct Sushi domain fold consisting of two antiparallel β sheets stabilized by a network of three intramolecular disulfide bonds and a salt bridge [6], [7]. Furthermore, similar to family A GPCRs, two highly conserved cysteine residues in extracellular loops ECL1 and ECL2 form an additional intramolecular disulfide bond. In the B1 family, the ECD-1 binds the C-terminal portion of the peptide ligand, while the extracellular loops and transmembrane domain interact with the N-terminal portion of the peptide ligand to initiate signaling [8].

Mammalian CRFRs bind endogenous corticotropin releasing factor (CRF) and urocortin peptides (Ucn1, Ucn2, and Ucn3), plus other natural CRF-related peptide ligands, such as amphibian sauvagines and fish urotensins, as well as synthetic compounds [9], [10]. CRF is a 41-amino acid (aa) peptide synthesized in the hypothalamus in the form of preprohormone [11]. A key player in the hypothalamic-pituitary-adrenal axis, CRF represents the primary activator of the central response to stress in mammalian organisms. CRF is produced by neuroendocrine cells of the paraventricular nucleus of the hypothalamus and is delivered via the hypothalamo-hypophyseal portal system to the anterior pituitary, where it regulates the secretion of the POMC gene, encoding several peptides, including ACTH. In addition, CRF is produced in other areas of the central nervous system and in a variety of peripheral tissues, where it acts as a neurotransmitter/neuromodulator. The urocortins [12], [13], [14] are also expressed in the central nervous system, as well as in many peripheral tissues. The anatomical distribution of CRF and the urocortins is distinct and these peptides are involved in a multitude of physiological mechanisms exerting complementary or contrasting actions.

In most vertebrates there are two highly conserved tissue-specifically expressed CRFRs, designated type 1 [15] and type 2 [16], encoded by different genes and approximately 70% identical to each other at the amino acid level. CRFR1 is relatively more abundant in the nervous system, including the pituitary, while CRFR2 is particularly expressed in the heart, skeletal muscle, and gastrointestinal tract. CRF has tenfold higher affinity for CRFR1 than for CRFR2; Ucn1 has similar affinity for both receptors; in contrast Ucn2 and Ucn3 are selective for CRFR2. The main type 1 isoform in human, rat and mouse is CRFR1α consisting of a 415 aa protein including a 23 aa cleavable signal peptide (SP) [17] and a 98 aa ECD-1. The human CRFR1α sequence is 97% identical to both rat and mouse. RNA alternative splicing generates many additional minor isoforms, which modulate the receptor activity [18]. Five putative conserved N-glycosylation sites (Asn: 38, 45, 78, 90, 98) [19] have been located in rat CRFR1α ECD-1 and three conserved disulphide bridges (Cys 30–54; 44–87; 68–102) have been predicted both in human [17] and rat [19] CRFR1α ECD-1s.

Mammals produce two main CRFR2 isoforms differing only at the far N-terminus. The CRFR2α variant is 411 aa long both in human and mouse, while the CRFR2β variant is 438 aa in human and 431 aa in mouse. Similar to CRFR1, three disulphide bridges (Cys 45–70; 60–103; 84–118) and five putative N-glycosylation sites have been predicted in mouse CRFR2β ECD [6]. In contrast to CRFR2β, which like CRFR1α has a canonical SP, the CRFR2α variant contains an unconventional pseudo SP [20].

The CRFRs display promiscuous signaling because of their ability to couple to multiple G-proteins and influence distinct intracellular networks in a tissue-specific way [21]. Such diversity of physiological actions makes these receptors increasingly important as drug targets.

In most cases, obtaining a sufficient amount of a purified membrane protein for biophysical studies requires a heterologous expression system. In this respect, production of recombinant GPCRs relies on bacterial, yeast, eukaryotic cells or cell-free methods, each method presenting its own advantages [22], [23]. The *E. coli* host, utilized in the present work, lacks endogenous G-proteins, necessary for GPCR signaling, but has great potential due to its simplicity, growth at high density, possibility to scale up, safety, and low cost. It can also employ the power of bacterial genetics with different strains and expression vectors. Most bacteria lack post-translational modifications, such as glycosylation, which may be critical for GPCRs function in eukaryotic cells, but also have the advantage of yielding homogeneous samples for structural investigations. Finally, bacteria allow for easy stable isotope labeling for protein NMR studies. Recombinant GPCRs expressed in *E. coli* can be obtained by producing the protein in a membrane-integrated form followed by detergent solubilization. Although limited in part by differences, such as presence of cholesterol, between the bacterial and the eukaryotic cell membrane, the *E. coli* expression system allows the GPCR to fold and assemble in an environment relatively similar to the native one. Mammalian GPCRs produced in *E. coli* membranes include β_2_-adrenergic receptor [24], [25], [26], serotonin 5-HT1A receptor [27], neurotensin receptor [28], [29], Nk-2 receptor [30], M1 muscarinic acetylcholine receptor [31], M2 muscarinic acetylcholine receptor [32], adenosine A_2A_ receptor [33], CB1-CB2 cannabinoid receptors [34], [35], [36] and CCR5-CCR3-CXCR4-CX3CR1 chemokine receptors [37]. A recent study has described the expression of numerous GPCRs in *E. coli* membranes [38]. Despite its promise, GPCR expression in *E. coli* membrane is highly unpredictable, i.e. dependent upon the particular GPCR, and often results in a very low yield. Bacterial overexpression of GPCRs can also result in incorrect folding and protein aggregation in the form of cytoplasmic inclusion bodies. These have limited toxicity to the bacterial host and may protect the protein from lysis. Therefore, as an alternative to membrane insertion, a second GPCR production strategy in bacteria consists of directing recombinant product into inclusion bodies, followed by protein denaturation and refolding [39], . However, it is to be taken into account that GPCR refolding might present challenges and that not all GPCRs expressed in *E. coli* produce inclusion bodies.

Several class B1 ECD-1 3D-structures, including CRFR ECD-1s, both in isolation or in complex with protein ligands have been determined [6], [7], [43], [44], [45]. Importantly, the class B1 structures of the trans-membrane domains of CRFR1α (aa 104–373) [46] and of glucagon receptor (GCGR) (aa 123–432) [47] have been recently determined. In order to promote functional analyses and structural studies we have undertaken the characterization of recombinant human CRFR1α (hCRFR1α) and mouse CRFR2β (mCRFR2β) produced in bacterial membranes. Using a series of expression plasmids, we verified the impact of different strains, culture media and protein SPs. Detergents were screened to optimize the solubilization of the receptors from the bacterial membranes. The functionality of the receptors expressed in *E. coli* intact cells and corresponding membrane fractions was assessed by radioreceptor assays using selected CRF family ligands.

## Results

### mCRFR2β transmembrane topology in E. coli

**Figure 1:**
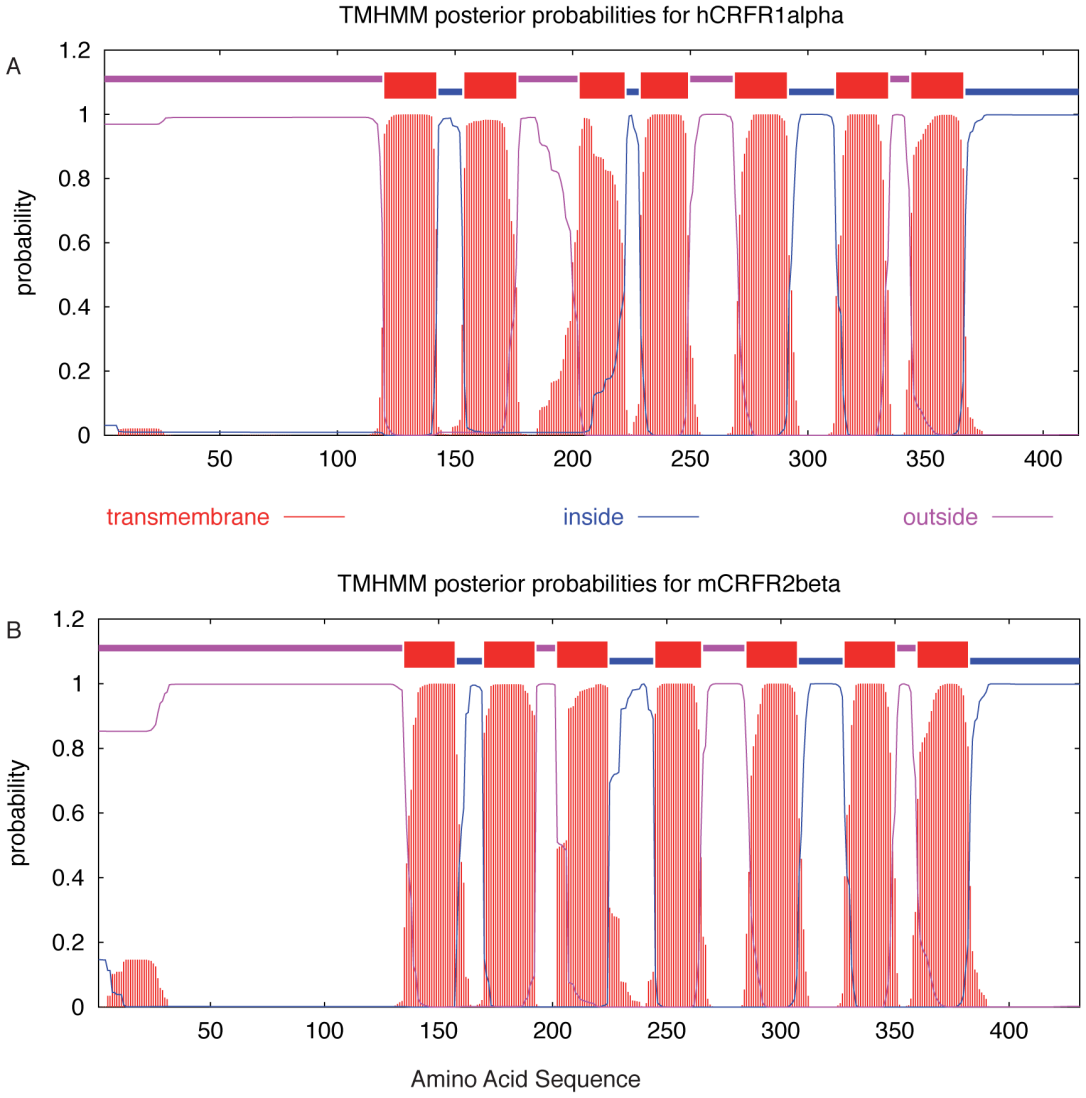
Transmembrane topology models applied to hCRFR1α and mCRFR2β. TMHMM analyses of the receptors' sequences. Red regions indicate putative transmembrane domains with the relative probability of each indicated on the Y-axis. Pink and blue regions correspond to predicted extracellular and intracellular segments respectively.

**Figure 2:**
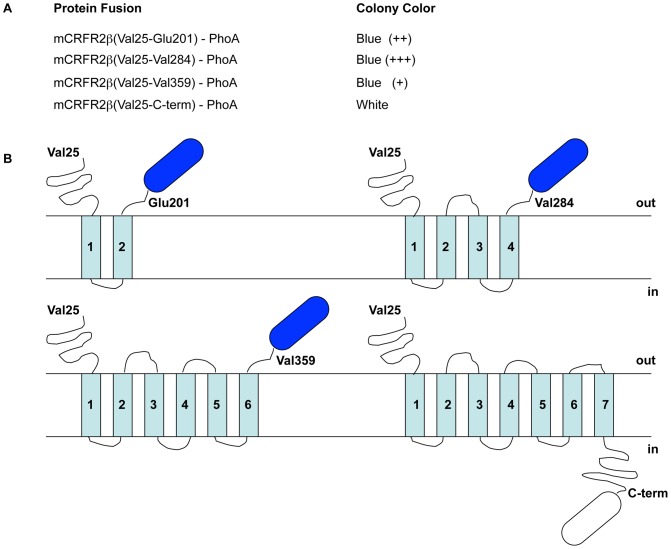
Alkaline phosphatase fusion protein analysis in *E. coli* of mCRFR2β. (**A**) Specifically designed C-terminally truncated versions of mCRFR2β fused to bacterial membrane topology reporter alkaline phosphatase (PhoA) confer different phenotypes at the level of colony color. PhoA activity was assessed qualitatively by visual inspection of the colonies. (**B**) The bacterial colony colors conferred by these different protein fusions are in agreement with the hepta-helical transmembrane model of mCRFR2β. The aa numeration refers to the native receptors pre-protein sequence.

The topology of an integral membrane protein predicts the number and sequence location of the membrane spanning segments, and their orientation relative to the biological membrane. CRFRs are predicted to have the topology typical of the GPCR superfamily. As a first step toward recombinant expression of the receptors in *E. coli*, a membrane topology model was derived for both receptor sequences hCRFR1α and mCRFR2β by means of the Trans Membrane Hidden Markov Model (TMHMM) [48]. The predictions confirm the classic seven transmembrane domain structures with a relatively large N-terminal ECD located outside and the C-terminus situated inside the cell (Figure 1). In an initial set of experiments we probed the transmembrane topology assumed by the receptors in the *E. coli* cell by designing C-terminally truncated versions of mCRFR2β fused to bacterial alkaline phosphatase (PhoA). The activity of this enzymatic reporter depends on its subcellular location and is only functional if exported into the periplasmic space [49], [50]. We constructed gene fusions by PCR attaching PhoA to the C-terminal extremity of the predicted first, second, and third extracellular loop and also to the receptor C-terminus. The original SP present in the mCRFR2β was not included in any of these gene fusions. PhoA activity was estimated qualitatively at the level of bacterial colony in a *phoA^−^ E. coli* strain (CC118) using the chromogenic substrate 5-bromo-4-chloro-3-indolyl phosphate (BCIP), which turns blue when hydrolyzed. As predicted by the topology model, bacteria producing each of the three protein fusions at the level of the extracellular loops displayed distinct PhoA activity. In addition, bacterial colonies carrying the full-length mCRFR2β-PhoA fusion remained white, which is consistent with the expected intracellular localization of the receptor C-terminus (Figure 2). Similar results were obtained when the mCRFR2β sequences included the PelB bacterial SP at the receptor N-terminus (data not shown).

### Bacterial expression vector design

Given that out of all GPCRs, an endogenous SP sequence is present primarily in that minority group, such as class B1 CRFRs, characterized by a long ECD-1 [51], we intended to determine the impact of a bacterial SP on our recombinant expression system. In *E. coli*, in contrast to eukaryotic systems, which usually display a co-translational mechanism, most soluble proteins secreted in the periplasm contain a canonical SP (such as the one present in the proteins PhoA, MalE) and are translocated in a post-translational manner, *via* the Sec-dependent pathway. In this case, the growing polypeptide chain is maintained in an unfolded status primarily by chaperone SecB. To this class belongs also the protein PelB of *Ervinia carotovora* (a Gram negative bacterium related to *E. coli*), whose SP is frequently used to direct recombinant proteins in the *E. coli* periplasmic space. On the contrary, the transmembrane segments of *E. coli* inner membrane-integrated proteins, plus a small fraction of soluble periplasmic proteins characterized by highly hydrophobic signal sequences, such as the one present in the protein DsbA [52], are translocated via the SRP-dependent pathway, which follows a co-translational mechanism. In light of the possibility that overexpression of long N-tail GPCRs, such as the two CRFRs, might be facilitated by either one of the two bacterial translocation mechanisms, we set to test the gene expression of both receptors fused to either PelB SP or DsbA SP and compared them to the CRFRs' expression in absence of SP. In all cases the endogenous receptor's SP was eliminated from the coding sequence.

**Figure 3:**
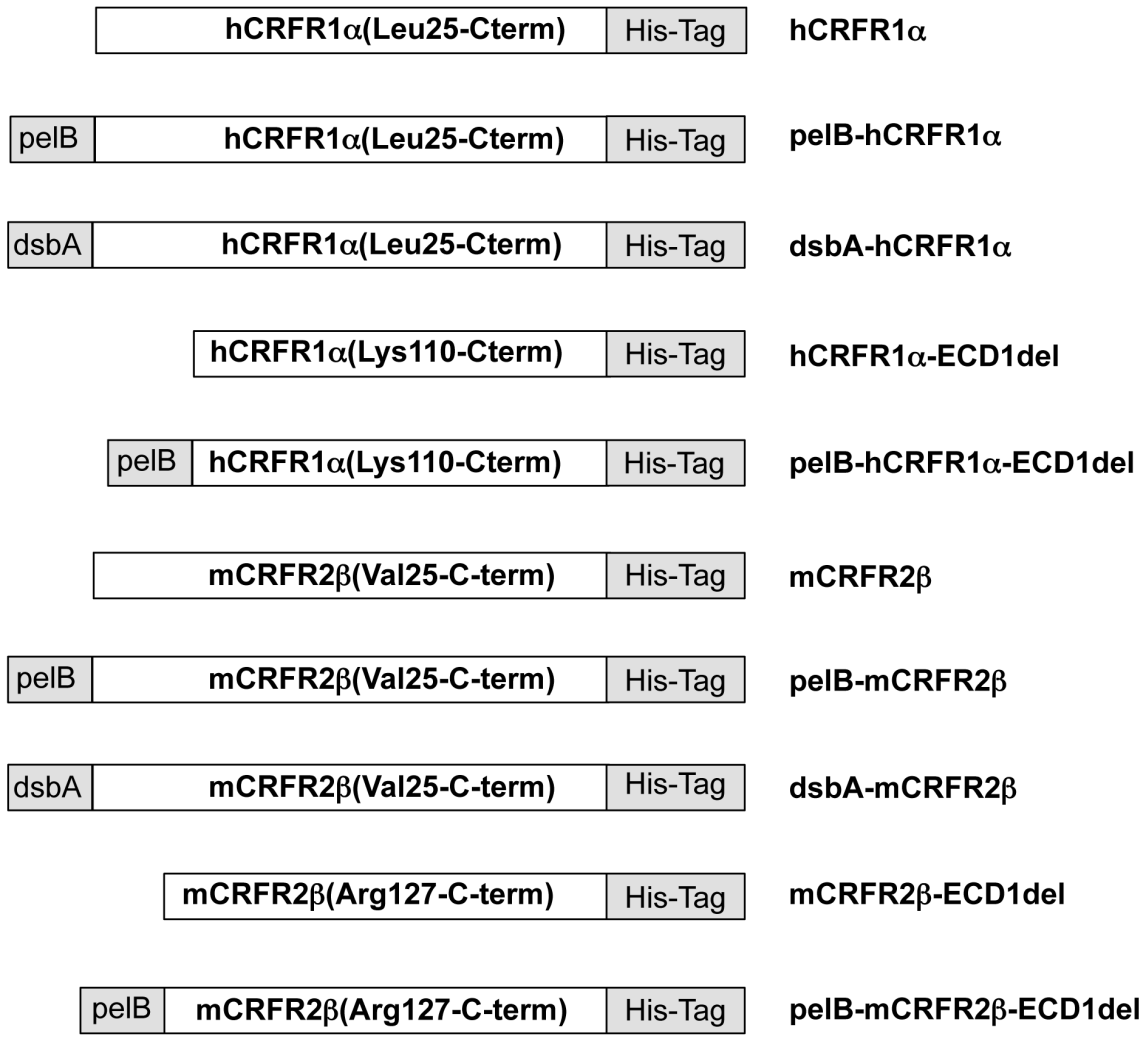
Schematic diagram of the fusion proteins used for the expression of CRFRs. The constructs differ by the presence of a bacterial signal peptide (PelB, DsbA, or none) and do not include the native signal peptide. They encode either full-length receptors or variants lacking the ECD-1 domain. All the proteins are produced with a His_6_-tag at the C-terminus. The aa numeration refers to the native receptors pre-protein sequences.

The expression vectors used in this study (Figure 3) were derived from the pET vector system, which provides a strong inducible promoter. To facilitate the optimization process, all the constructs had an identical backbone, carrying the T7lac promoter and the kanamycin resistance marker. The latter was chosen over ampicillin, since it confers better plasmid stability and because the expression of the kanamycin resistance gene does not involve the bacterial secretory pathway. The cDNAs encoding full-length mature forms of hCRFR1α and mCRFR2β were introduced into pET-26b plasmid bearing the N-terminal PelB SP. Two vector derivatives were used, wherein the DsbA SP replaced PelB SP, plus two similar vectors lacking a SP altogether. In addition plasmids encoding either hCRFR1α or mCRFR2β, which lack the N-terminal ECD, were built in two versions: with or without the PelB SP. All plasmids encoded a His_6_-tag at the C-terminus separated from the GPCR cDNA by the same sequence (NSSSVDKLAAALE), which is part of the pET-26b original expression vector.

### Influence of E. coli host strains

The selection of parameters for the production of recombinant proteins remains an empirical process. Initially, we evaluated the expression of full-length PelB-hCRFR1α and PelB-mCRFR2β in the widely used *E. coli* BL21(DE3), which is deficient in *lon* protease and lacks *ompT* outer membrane protease, and carries the gene for T7 RNA polymerase under the *lac*UV5 promoter on the *E. coli* chromosome. Bacterial cultures were grown in 500 ml Luria Bertani (LB) broth at 37°C up to an OD_600_ of 0.9, transferred at 18°C and shortly after induced with 1 mM IPTG for 24 h. Next we tested under the same “standard” expression protocol the BL21(DE3) derivative strains C41(DE3) and C43(DE3), which carry a mutation promoting the expression of toxic recombinant proteins [53], and strain Rosetta2(DE3), which supplements tRNAs for seven *E. coli* rare codons.

**Figure 4:**
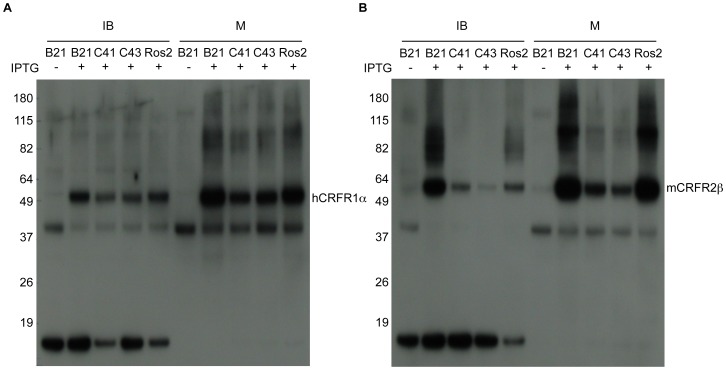
Influence of various *E. coli* host strain on the expression of CRFRs. Expressions of PelB-hCRFR1α (**A**) and PelB-mCRFR2β (**B**) were carried out in LB medium in four different strains. Equivalent volumes of the bacterial inclusion bodies and membrane fractions were analyzed by Western blot with His_6_-tag antibody. Parental strain BL21(DE3) transformed with the same constructs but treated without IPTG inducer was used as a negative control.

Bacterial cells were collected and subjected to lysis and fractionation. Equivalent volumes of endpoint cultures were processed. These do not necessarily correspond to equal number of cells. Cell debris and inclusion bodies were pelleted with a low speed centrifugation step. This fraction was denoted IB. Membranes (M) were separated from the soluble fraction via high-speed centrifugation. IB and M fractions were analyzed by SDS-PAGE and Western blot using a polyclonal anti-His_6_-tag antibody (Figure 4). As a negative control we used bacteria transformed with PelB-CRFRs but lacking the IPTG inducer. Interestingly, in the tested conditions, across all the examined bacterial hosts most of recombinant receptor was inserted in the bacterial membrane. Overall, hCRFR1α was recovered at slightly higher level in strain C43(DE3) than in strain C41(DE3), while the opposite was observed with mCRFR2β. However the level of recombinant receptors with these two strains, was substantially lower in comparison to the parental strain BL21(DE3). The latter, along with derivative Rosetta2(DE3), produced the highest amount of recombinant receptors and were selected for subsequent experiments (Figure 4). On the Western blot we noted the presence of two series of bands (one restricted to the IB fractions, and the other prevalent in the M fractions) representing proteins of lower molecular weight relative to the CRFRs. These bands appear to be due to other proteins cross-reacting with the anti-His_6_-tag Ab as opposed to N-terminal degradation products of the CRFR proteins, because they are also present in the non-induced protein preparations. Because of the location of the His_6_-tag, our Western analysis cannot detect any potential partial length product, which may be the result of a C-terminal deletion.

### Receptors' relative expression level

**Figure 5:**
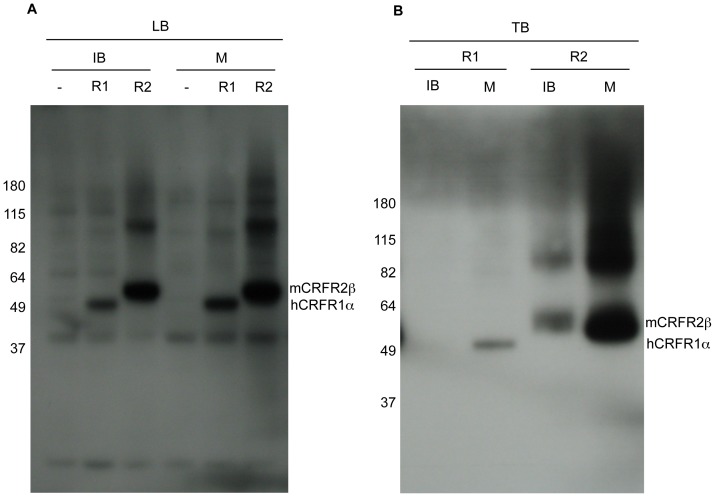
Comparison of the expression level of recombinant CRFRs. Expression of PelB-hCRFR1α (R1) and PelB-mCRFR2β (R2) was carried out either in LB (A) or in TB (B) medium in Rosetta2(DE3) strain. TB derived cultures were diluted twenty times before electrophoresis, while LB derived fractions were not diluted. With either medium, equivalent volumes of both the bacterial inclusion bodies and membrane fractions were analyzed by Western blot with His_6_-tag antibody. IPTG induced bacteria Rosetta2(DE3) transformed with the parental vector pET-26b (-) were used as a negative control.

Next we directly compared by Western blot analysis the expression level in LB medium of the two recombinant receptors produced in strain Rosetta2(DE3). The bacterial fractions derived from the PelB-mCRFR2β construct showed more recombinant receptor protein in comparison to the equivalent fractions derived from the PelB-hCRFR1α plasmid (Figure 5, A). As a negative control we used bacteria transformed with the “empty” parental vector pET-26b, supplemented with IPTG. The position of the recombinant receptor bands was found to be in good agreement with their expected molecular mass, which is 47.5 kDa for the mature form of PelB-hCRFR1α and 49.5 kDa for the mature form of PelB-mCRFR2β. Another band of approximately 100 kDa molecular mass is indicative of the presence of receptor dimers, which, in the tested conditions, appear to be resistant to a complete denaturation (Figure 5, A).

### Influence of E. coli culture medium

When the bacteria were grown in TB medium, we also observed, similar to LB medium, a higher expression level of mCRFR2β compared to hCRFR1α (Figure 5, B). Replacing LB with TB culture medium is a standard approach to increase bacterial biomass. The use of TB enhanced the protein level of both recombinant receptors by at least five times, as demonstrated by Western blot analysis in which undiluted fraction samples derived from LB cultures were directly compared to five-fold dilutions of fraction samples derived from TB cultures (data not shown). It was also found that, with TB medium in the strain Rosetta2(DE3), a slightly lower level of recombinant receptors was detected in the IB fractions in comparison to parental strain BL21(DE3) (Figure S1). At least in the case of the type 1 receptor, expression in the tRNAs supplementing strain corresponded also to a higher level of the GPCR product in the M fraction (Figure S1). Based on these results Rosetta2(DE3) was identified as the *E. coli* strain of choice for the production of membrane-integrated CRFRs. In addition, to show the reproducibility of our protocol, the Western blot results of two independent expression experiments (starting from separate vector DNA transformations) relative to PelB-hCRFR1α and PelB-mCRFR2β in Rosetta2(DE3) grown in TB medium are reported (Figure S1).

**Figure 6:**
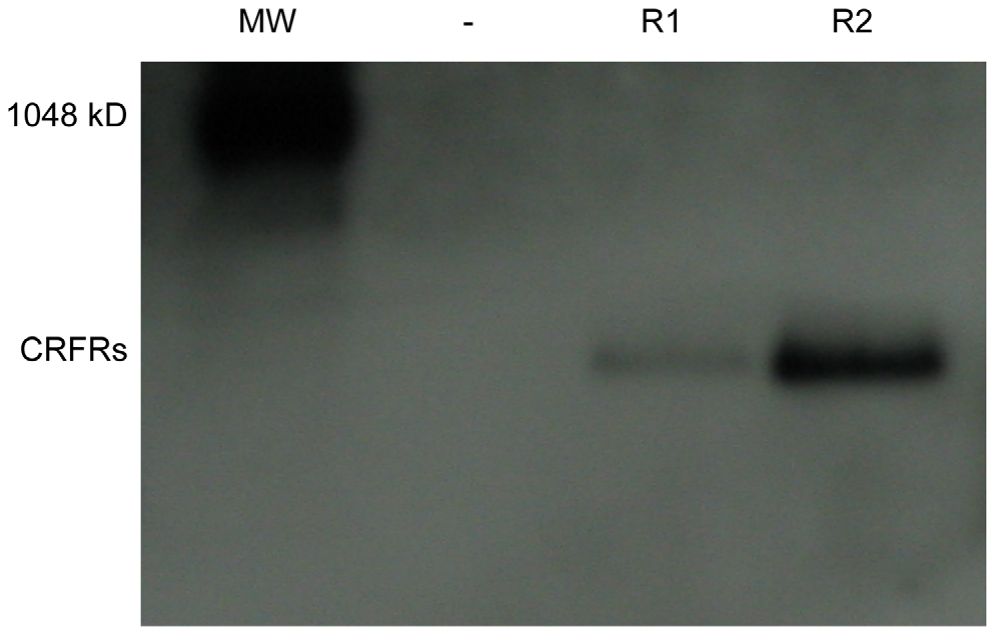
Native Western blot analysis of bacterial membrane fractions. Native PAGE Western blot analysis with anti-His_6_-tag antibody of detergent-free bacterial membrane fractions, which derived from the expression of PelB-hCRFR1α (R1) and PelB-mCRFR2β (R2) carried out in Rosetta2(DE3) strain and TB medium. Vector pET-26b (-) was used as negative control. The 1,048 kDa band corresponds to the smallest protein (IgM pentamer) present in the NativeMark Unstained Protein Standard (Life Technologies), which is detected by the antibody.

To further investigate the status of the receptors localized in the *E. coli* membrane, aliquots of the M fractions were subjected to native PAGE Western blot analysis with anti-His_6_-tag antibody. Membranes derived from PelB-mCRFR2β in Rosetta2(DE3) strain and TB medium display a single clear band corresponding to an oligomeric structure (Figure 6). In comparison, PelB-hCRFR1α samples display a less intense band, consistent with the lower accumulation level previously observed of the type 1 receptor but indicative of a structure of similar size (Figure 6). No band was observed for membranes originating from the “empty” vector pET-26b, which was used as negative control. While the composition and the stoichiometry of these native assemblies within the bacterial membrane remain to be determined, their occurrence might be exploited in a strategy toward the purification of the receptors in absence of detergents.

### Effect of the signal peptides

**Figure 7:**
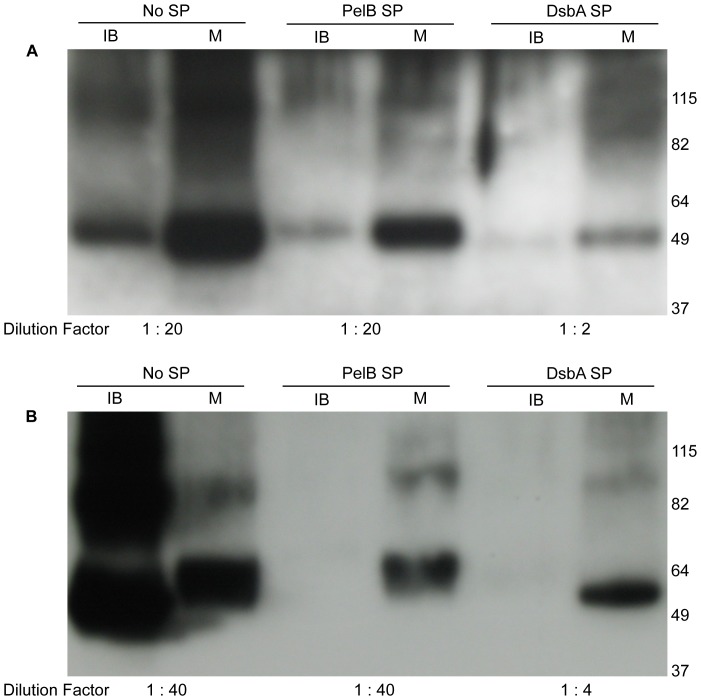
Influence of signal peptides on the expression of CRFRs. Comparative analysis of hCRFR1α (A) and mCRFR2β (B) constructs encoding no signal peptide (No SP), PelB signal peptide or DsbA signal peptide. Expressions were carried out in TB medium with Rosetta2(DE3) strain and the samples were analyzed by Western blot with His_6_-tag antibody. For each expression vector tested, 1 µl of IB and M fractions were loaded on the gel; the dilution factor of each sample is indicated.

Additional experiments were carried out to evaluate the impact of the SP on the production of recombinant CRFRs. DsbA-hCRFR1α and DsbA-mCRFR2β constructs were transformed in Rosetta2(DE3) and grown in TB medium. The proteins were produced with the protocol described above and identified by Western blot analysis, which again indicated a higher yield for the type 2 relative to the type 1 receptor (Figure S2). Next, a direct comparison of the human type 1 receptor level expressed with either PelB, or DsbA, or no SP was carried out. Replacing PelB SP with DsbA SP resulted in a marked drop in the receptor expression in both the IB and the M fractions, while eliminating SP resulted in increased level in the above fractions (Figure 7, A). A similar trend was observed in the case of the recombinant mCRFR2β (Figure 7, B). In this case the removal of the SP resulted in a higher expression level in the M fraction, together with a major increase of the product recovered in the IB fraction. In summary, in the tested conditions, with regard to the yield of full-length membrane-integrated CRFRs, the following ranking scale could be determined in connection with the effect of the SPs: No SP>PelB SP≫DsbA SP.

### Expression of CRFRs variants lacking ECD-1

**Figure 8:**
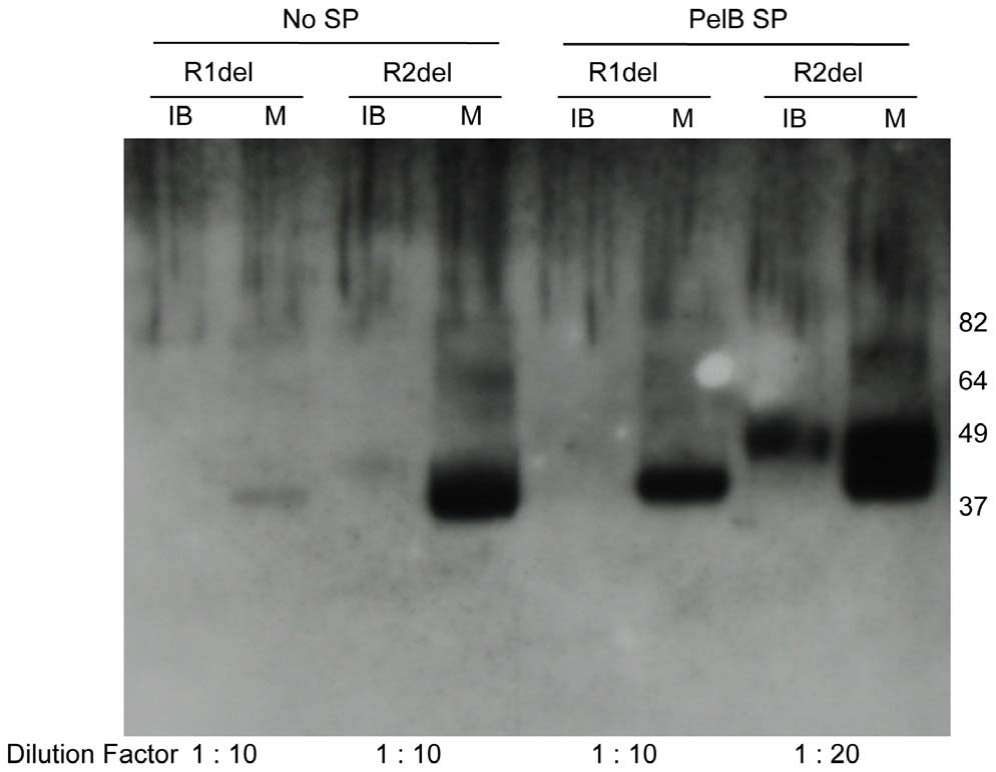
Expression of CRFRs variants lacking ECD-1. Comparative analysis of hCRFR1α (R1del) and mCRFR2β (R2del) constructs lacking the receptor N-terminal ECD-1 and encoding either no signal peptide (No SP), or PelB signal peptide (PelB). Expressions were carried out in TB medium and Rosetta2(DE3) strain and the samples were analyzed by Western blot with His_6_-tag antibody. For each expression vector tested, 1 µl of IB and M fractions was loaded on the gel; the dilution factor of each sample is indicated.

Eliminating the ECD-1 reduces the target sequence length by approximately 100 aa, about one fourth of the full-length protein sequences. We have expressed the CRFRs lacking their N-terminal ECDs, with or without PelB SP. The constructs hCRFR1α-ECD1del and mCRFR2β–ECD1del as well as equivalent PelB constructs PelB-hCRFR1α-ECD1del and PelB-mCRFR2β–ECD1del were all expressed in Rosetta2(DE3) bacteria grown in TB medium and fractionated with the standard protocol. Western blot analysis with anti-His_6_-tag antibody detected a band for both receptor deletion mutants in good agreement with the expected 38 kDa molecular mass. The great majority of the N-terminally truncated receptors were found in the bacterial membrane fractions, and again substantially more type 2β receptor was present in comparison to the type 1α (Figure 8). In addition, in the case of hCRFR1α, the presence of the PelB SP resulted in a clear increase in the level of the membrane-integrated form of truncated receptor.

### Solubilization of CRFRs from membrane fractions

**Figure 9:**
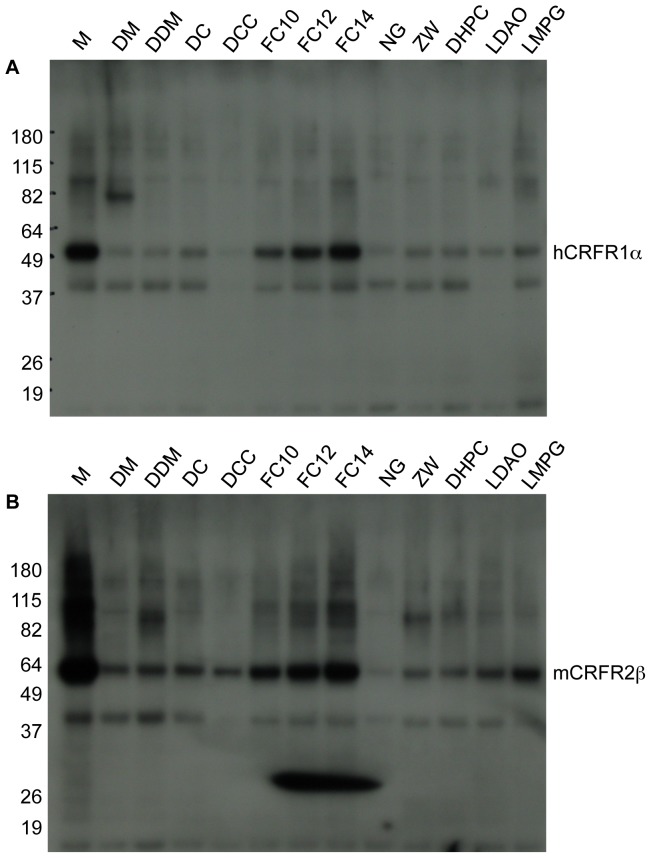
Detergent screen for solubilization of CRFRs. The efficacy of 12 different detergents, or detergent mixes, in solubilizing PelB-hCRFR1α (**A**) and PelB-mCRFR2β (**B**) from bacterial membranes was evaluated, after overnight incubation, by phase separation via ultracentrifugation. The resultant soluble fractions were subjected to His_6_-tag antibody Western blot analysis. As a control, an equivalent aliquot of the original membrane fraction (M), which had not been subjected to solubilization, was loaded on the gel. The irregular spot present in the lower part of the B panel is due to a non-specific contamination. For abbreviations of detergent molecules see main text.

Purification of GPCRs requires the extraction of the receptors from the membranes with detergents. The properties of the detergent are key for both an effective extraction and to preserve the structure and function of the receptor proteins; choice of the detergent is to a large extent an empirical process. Aliquots of the M fractions derived from PelB-hCRFR1α and PelB-mCRFR2β produced in Rosetta2(DE3) strain and with LB medium, were tested for solubilization with different detergent molecules. These were DM, DDM, FC-10, FC-12, FC-14, NG, ZW-3.12, DHPC, LDAO, LMPG (for abbreviations see Materials and Methods) each used at a single concentration well above the individual specie's critical micelle concentration (CMC). Two mixtures were also tested: DC (DDM+CHS) and DCC (DDM+CHS+CHAPS). After overnight extraction at 4°C the samples were subject to ultracentrifugation and the supernatant was analyzed by Western blot with anti-His_6_-tag antibody (Figure 9). As a control, an aliquot of untreated M fraction was also loaded on the gel. Out of the twelve conditions tested, FC-10, FC-12, and particularly FC-14 accomplished the better extraction for both CRFRs. In subsequent experiments we investigated the effect of FC-14 at concentrations between 5 mM and 25 mM; within this concentration range there was no difference in the amount of protein solubilized (data not shown).

### Functional Characterization: Receptor-Ligand Binding

**Figure 10:**
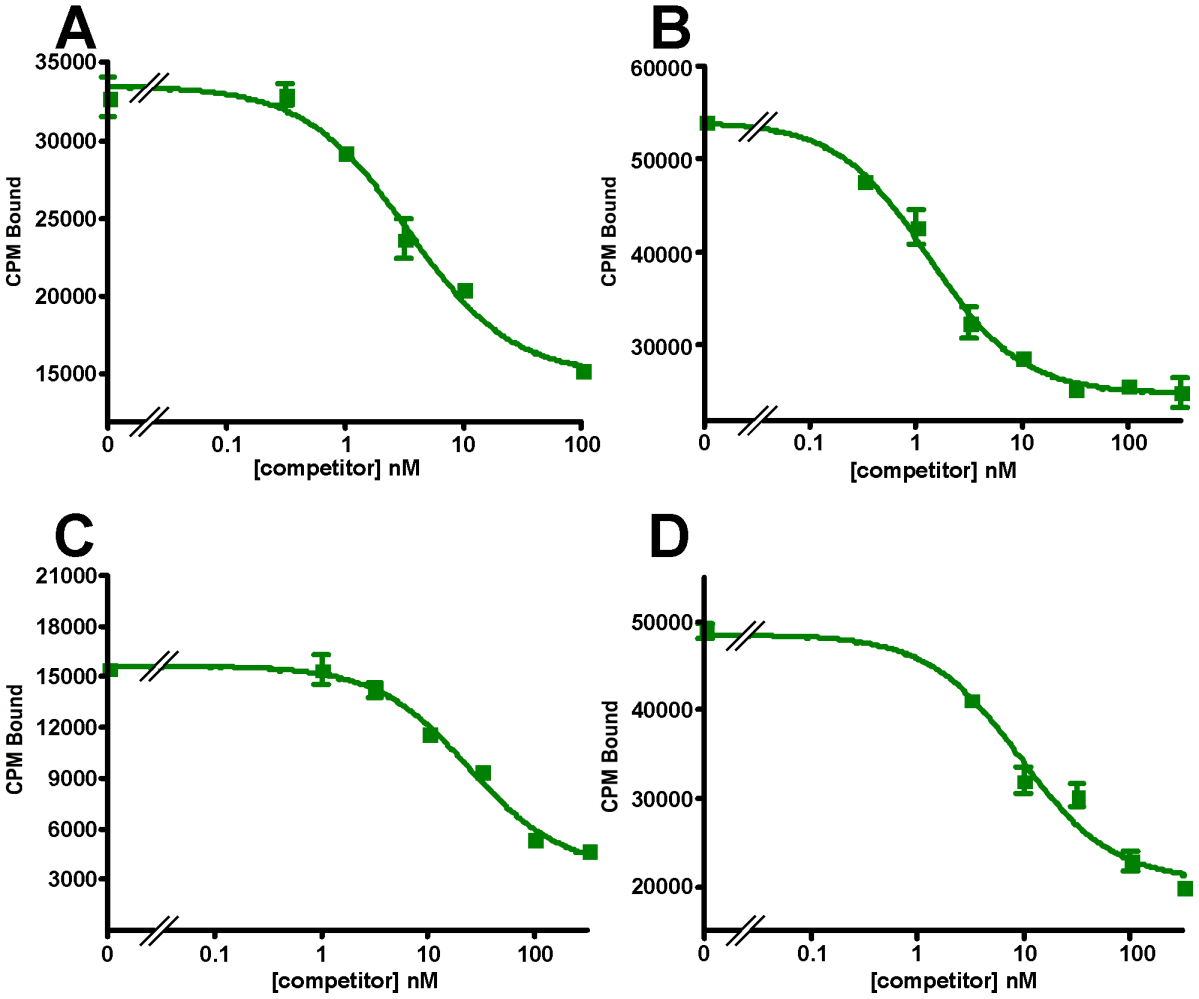
Astressin binding. Displacement by astressin (◼) of labeled astressin bound to, (A) hCRFR1α, (B) mCRFR2β expressed in *E. coli* membranes, or (C) hCRFR1α, (D) mCRFR2β expressed in *E. coli* spheroplasts.

**Figure 11:**
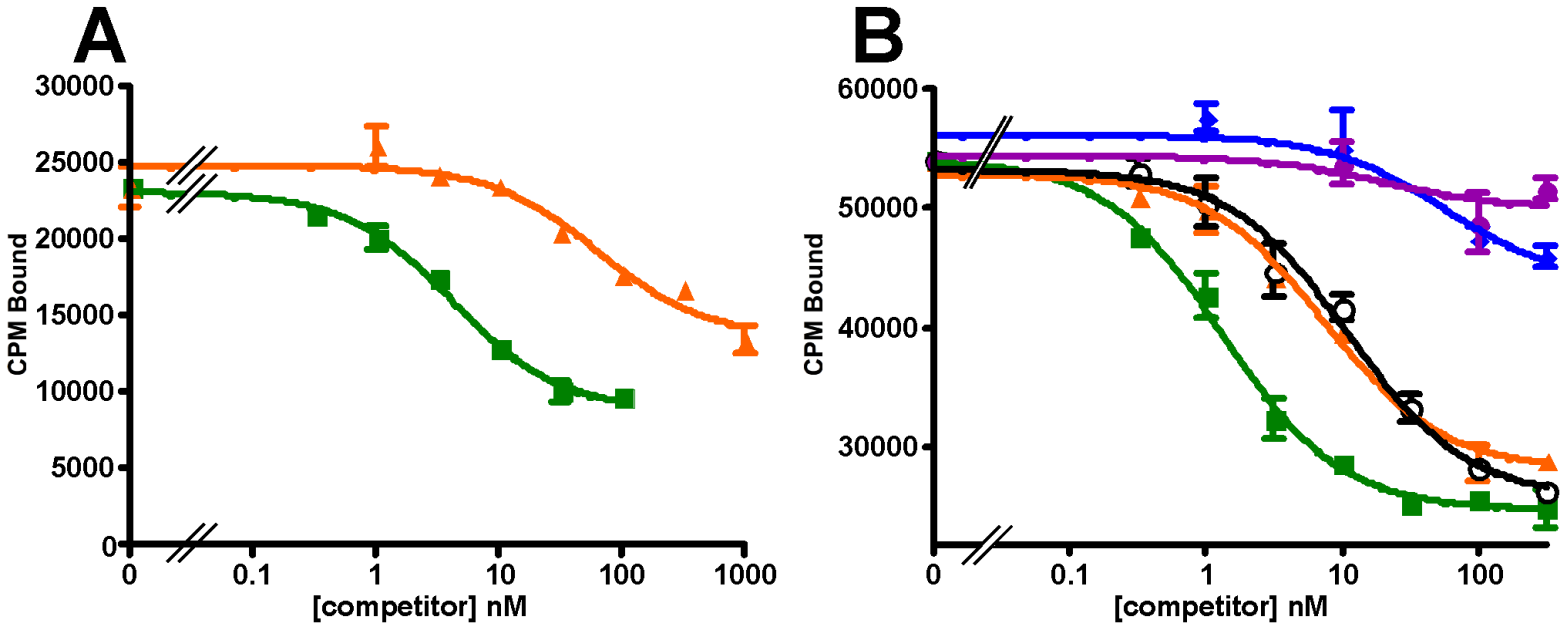
Specificity of astressin binding. Displacement by astressin (◼), Ucn1 (▲), Ucn2 (◯); Ucn 3 (♦) or sauvagine (●) of labeled astressin bound to (A) hCRFR1α or (B) mCRFR2β expressed in *E. coli* membranes.

**Figure 12:**
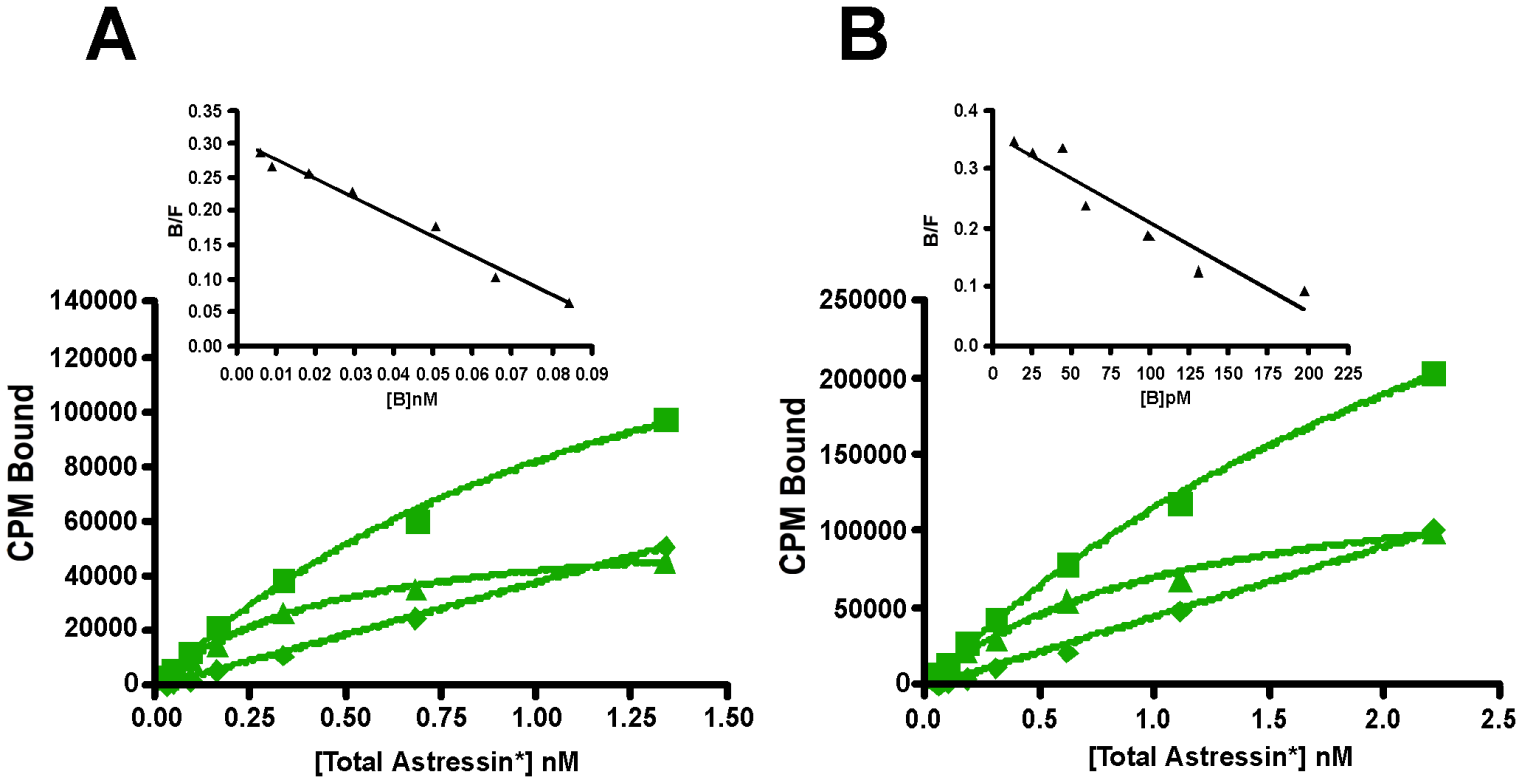
Saturation binding of labeled astressin. Binding of increasing concentrations of labeled astressin bound to (A) hCRFR1α or (B) mCRFR2β expressed in *E. coli* membranes. (◼) total binding; (♦) non-specific binding; (▲)specific binding.

To verify the functionality of the recombinant receptors we evaluated their ability to bind selected ligands, including astressin, CRF, Ucn1, Ucn2, Ucn3, sauvagine, PD-sauvagine, and antalarmin. PD-sauvagine is a new CRF receptor ligand, secreted by the skin of Mexican leaf frog *Pachymedusa dacnicolor*, whose N-terminal half is similar to CRF while its C-terminal half is similar to sauvagine [54]. Both receptors expressed in either *E. coli* intact cells or their membrane fractions bind the antagonist astressin with nanomolar affinity (Figure 10). Sauvagine does not displace labeled astressin bound to either receptor. The receptors also fail to bind labeled sauvagine. Ucn1 competitively displaces labeled astressin bound to both receptors, while the CRFR2-selective ligand, Ucn2, competitively displaces labeled astressin bound to mCRFR2β but not to hCRFR1α; the other CRFR2-selective ligand Ucn3 does not compete for binding to mCRFR2β (Figure 11). The observation that Ucn1 does not completely displace all bound counts on hCRFR1α suggests that there are additional sites to which astressin binds that are not accessible to Ucn1. The results for the ligands are summarized in Table 1. The K_i_ values for the CRF ligands are comparable, but not precisely the same as those found for receptors expressed in mammalian cells [14]. The K_d_'s for labeled astressin bound to either receptor were obtained from saturation experiments shown in Figure 12; K_d_ = 0.59 (0.39–0.88) nM for hCRFR1α and K_d_ = 1.1 (1.0–1.3) nM for mCRFR2β (n = 2).

**Table 1:** Inhibitory binding constants (K_i_, nM) for CRF ligands bound to CRFRs expressed in *E.coli* membranes as measured by competitive displacement of bound labeled astressin.

Receptor	Astressin	rUcn1	mUcn2	mUcn3	r/hCRF	PD-Sauvagine*
CRFR1α	2.6 (1.5–4.6) (n = 14)	98 (57–167) (n = 6)	N.D.	N.D.	N.D.	5.3 (1.7–16.6) (n = 5)
CRFR2β	4.9 (2.3–10.3) (n = 8)	11.5 (4.0–32.7) (n = 3)	17.7 (11.3–27.6) (n = 3)	N.D.	N.D.	4.3 (2.9–6.5) (n = 2)

K_i_ values determined by analysis using competitive displacement of labeled astressin as described in Materials and Methods. r: rat; m: mouse; h: human. N.D.: No displacement.

* Inhibitory binding constants (K_i_, nM) for PD-sauvagine bound to CRFRs expressed in *E. coli* membranes were measured by competitive displacement of bound labeled PD-sauvagine.

**Figure 13:**
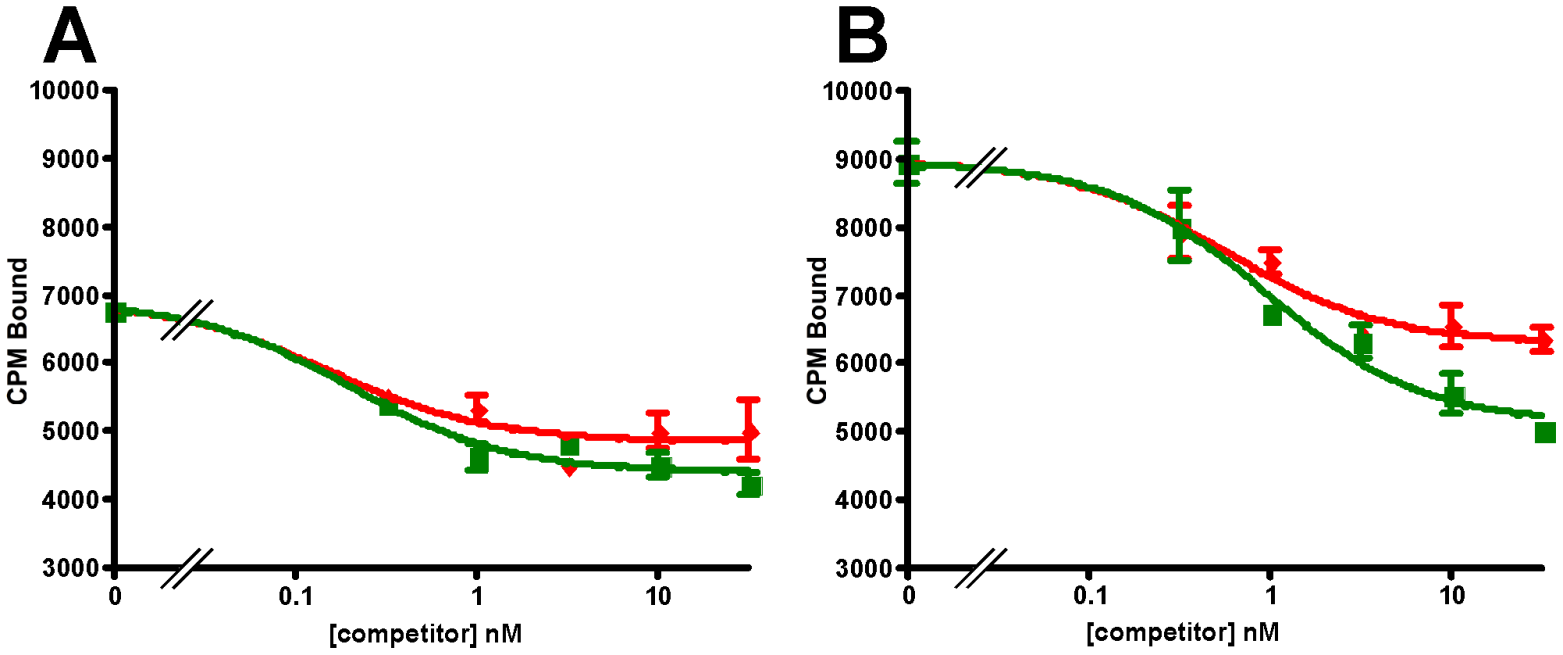
PD-sauvagine binding. Displacement by PD-sauvagine (♦) or astressin (◼) of labeled PD-sauvagine bound to (A) hCRFR1α or (B) mCRFR2β expressed in *E. coli* membranes.

Consistent with the competitive displacement by Ucn1 of bound labeled astressin, we have also observed binding of labeled Ucn1 to the receptors (data not shown). Interestingly, while the receptors in the *E. coli* membrane fractions do not bind the labeled agonist sauvagine, they do bind the other labeled agonist, PD-sauvagine (Figure 13). The number of sites detected by labeled PD-sauvagine is less than that detected by labeled astressin [55]. The K_d_'s for labeled PD-sauvagine were not determined because at nanomolar concentrations the binding did not saturate (Figure S3).

**Figure 14:**
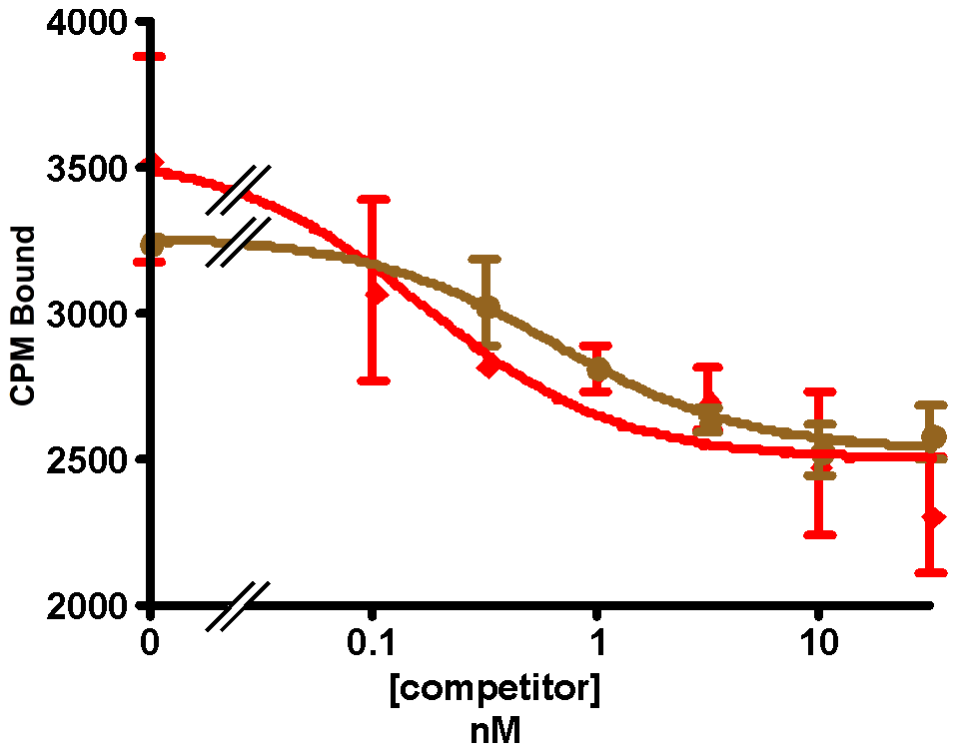
PD-sauvagine binding. Displacement by PD-sauvagine (♦) or antalarmin (●) of labeled PD-sauvagine bound to hCRFR1α expressed in *E. coli* membranes.

Interestingly, antalarmin, a CRFR1-selective small molecule antagonist, displaces labeled PD-sauvagine bound to hCRFR1α (Figure 14) in a dose dependent manner, similar to its displacement of labeled PD-sauvagine bound to hCRFR1α expressed in mammalian cell membranes (Perrin et al., to be published).

## Materials and Methods

### Bacterial strains, plasmids and oligonucleotides

The following are the *E. coli* bacterial strains used in this study. For transmembrane topology assay: CC118 [*araD139* Δ*(ara,leu)7697* Δ*lacX74 phoA*Δ*20 galE galK thi rpsE rpoB argE*_am_ *recA1*] (courtesy of Dr. Colin Manoil). For recombinant protein production: BL21(DE3) [F^−^ *ompT hsdS_B_ (r_B_^−^ m_B_^−^) gal dcm* (DE3)] (Novagen), OverExpress C41(DE3) and C43(DE3), which are derivative of BL21(DE3) containing genetic mutations phenotypically selected for conferring tolerance to toxic proteins (Lucigen), and Rosetta2(DE3) [F^−^ *ompT hsdS*_B_(*r_B_^−^ m_B_^−^*) *gal dcm* (DE3) pRARE2 (Cam^R^)] (Novagen) providing tRNAs for seven *E. coli* rare codons. For DNA vectors cloning and propagation: One Shot® TOP10 (Life Technologies). Human cDNA for CRFR1α was amplified from DNA GenBank L23332. Mouse cDNA for CRFR2β was amplified from DNA GenBank U17858. Oligonucleotide sequences are listed in Figure S4.

The mCRFR2-PhoA gene fusions were obtained introducing the various length receptor sequences into a low copy number vector derivative of pACYC184, at the N-terminus of a signal peptide-lacking alkaline phosphatase gene controlled by the kanamycin gene promoter. The mCRFR2β sequences listed in Figure 2 were all amplified by PCR using the same forward oligo C8 and reverse oligos C4 for mCRFR2β(Val25-Glu201)-PhoA, C5 for mCRFR2β(Val25-Val284)-PhoA, C6 for mCRFR2β(Val25-Val359), and C7 for mCRFR2β(Val25-C-term). The PCR products were introduced into the above vector digested with SacI and SpeI.

The CRFRs' amino acid sequences present in each protein expression vector are indicated in Figure 3. Plasmids hCRFR1α, mCRFR2β, hCRFR1α-ECD1del, mCRFR2β-ECD1del were obtained by replacing the NdeI-EcoRI fragment of pET-26b (Novagen) with the PCR products amplified with oligos C29-C14, C30-C12, C31-C14, C32-C12 respectively. Plasmids PelB-hCRFR1α, PelB-mCRFR2β, PelB-hCRFR1-ECD1del, and PelB-mCRFR2β-ECD1del were derived by replacing the NcoI-EcoRI fragment of pET-26b with the PCR products amplified with oligos C13-C14, C11-C12, C33-C14, C34-C12 respectively. Plasmids DsbA-hCRFR1α and DsbA-mCRFR2β were derived from the corresponding PelB vectors by replacing the NdeI-NcoI fragment with the DNA cassette provided by oligos C27-C28. In all the expression vectors the receptors' sequence was confirmed by DNA sequencing.

### Membrane topology assays

CRFRs membrane topology information was derived from UniProtKB/Swiss-Prot protein knowledgebase and prediction of transmembrane helices was generated using TMHMM Server v. 2.0, Center for Biological Sequence Analysis at the Technical University of Denmark (www.cbs.dtu.dk/services/TMHMM/) [48].

PhoA activity was scored based on the colour of bacterial colonies after overnight growth at 37°C on LB chloramphenicol (25 µg/ml) plates containing BCIP 20 µg/ml (Sigma), followed by 24 h incubation at room temperature and 24 h incubation at 4°C.

### Overexpression of CRFRs in E. coli

In order to produce recombinant CRFR proteins, the expression vectors were always freshly transformed in the *E. coli* strain of choice and selected on LB plates with antibiotic kanamycin 50 µg/ml, plus, in the case of Rosetta2(DE3), chloramphenicol 25 µg/ml. Single colonies were inoculated in 6 ml of LB medium containing antibiotic. The next day, 2.5 ml of each culture were inoculated into 500 ml LB (or TB) (1∶200) plus antibiotic in 2.8 L flasks, and incubated at 37°C with shaking at 260 rpm. The cultures were continued until the OD_600_ reached 0.9. Next they were incubated for 15 min at 4°C and then returned to the shaker incubator for 15 min 18°C. Subsequently 1 ml of the inducer IPTG (Life Technologies) 0.5 M (final concentration 1 mM) was added to the cultures. The incubation was continued for exactly 24 h at 18°C with shaking at 260 rpm.

### Preparation of intact E. coli cells

Preparation of intact *E. coli* cells was conducted as described by Grisshammer et al. [28]. Bacteria producing CRFRs were grown with the above general expression protocol, except that the IPTG final concentration was 0.5 mM and the incubation time was 18 h. Collected bacteria were resuspended in 0.5 ml aliquots in LB plus glycerol 25% at a concentration of 3.3×10^10^ and stored at 80°C. Freshly thawed aliquots were centrifuged at 6,000 rpm at 10°C and the cells were resuspended in 50 mM Tris HCl pH 7.5, 1 mM EDTA, 0.1% BSA.

### Bacteria fractionation

End point cultures were collected and centrifuged for 15 min at 5,000 rpm at 6°C. Bacterial pellets were resuspended in 50 ml TN buffer (50 mM Tris HCl pH 8, 300 mM NaCl). The samples were supplemented with 50 mg lysozyme, from chicken egg white (Sigma L6876), and one Complete EDTA-free tablet (protease inhibitor cocktail, Roche 11873580001) and stirred for 30 min at 4°C.

The bacteria were sheared by passing them twice through a microfluidizer processor (M-110L Pneumatic, Microfluidics), with a final sample volume of approximately 65 ml. The lysed cells were centrifuged for 15 min at 10,000 rpm at 6°C. The pellet, containing cell debris and inclusion bodies, designated the IB fraction, was resuspended in 20 ml TNG buffer (50 mM Tris HCl pH 8, 300 mM NaCl, 25% glycerol; TNG buffer was prepared mixing 20 ml glycerol to 80 ml TN Buffer). The supernatant was further centrifuged for 90 min at 40,000 rpm at 6°C. The supernatant was discarded, while the pellet represents the membrane (M) fraction. This was resuspended in 20 ml TNG buffer and further homogenized by stirring overnight at 4°C. Both the IB and the M fractions were stored in 0.5 ml aliquots at −80°C.

### PAGE and Western blot analysis

To determine the level and subcellular localization of recombinant receptor in *E. coli*, sample fractions were subject to reducing SDS-PAGE using the NuPAGE electrophoresis and blotting system (Life Technologies). Samples were preheated at 70°C for 10 min in the presence of NuPAGE LDS sample buffer and 100 mM DTT, or NuPAGE reducing agent and applied to NuPAGE 12% Bis-Tris gels (1 mm thick) with MES SDS running buffer.

Proteins were transferred to Invitrolon PVDF membranes in 1× NuPAGE Transfer Buffer with 10% methanol at 30 V constant for 1 h. The membranes were pre-wetted in methanol, blocked in TBS-T buffer (Tris-buffered saline, 0.1% Tween-20) containing 5% non fat dry milk, and incubated with rabbit polyclonal Ab to His_6_-tag-HRP (Abcam ab1187) diluted 1∶5,000 in TBS-T. Membranes were developed using ECL chemiluminescence (Millipore).

Native PAGE Western blot analysis was conducted using NativePAGE Novex Bis-Tris Gel System and following the manufacturer's (Life Technologies) recommendations. Samples were run without the addition of detergent, without the use of the 5% G-250 sample additive and with the Light Blue Cathode buffer. The native gel (4–16%) was run at 150 V for 100 min at RT and transferred at 25 V for 1 h. The membrane was treated in 8% acetic acid for 15 min, followed as above by immunodetection with the His_6_-tag–HRP antibody.

### Detergent screening for receptors solubilization

For membrane solubilization, 0.7 ml M fraction samples in TNG buffer, each corresponding to approximately 17 ml of endpoint Rosetta2(DE3) LB cultures, were supplemented with different detergents in a total volume of 1 ml containing 1× protease inhibitor cocktail (Roche 11873580001). Detergents (Anatrace) and working concentration: DM (n-decyl-β-D-maltopyranoside) 20 mM, DDM (n-dodecyl-β-D-maltopyranoside) 15 mM, FC-10 (n-decylphosphocholine) 30 mM, FC-12 (n-dodecylphosphocholine) 20 mM, FC-14 (n-tetradecylphosphocholine) 20 mM, NG (n-nonyl-β-D-glucopyranoside) 20 mM, ZW-3.12 (n-dodecyl-N,N-dimethyl-3-ammonio-1-propanesulfonate) 20 mM, DHPC (1,2-dihexanoyl-*sn*-glycero-3-phosphocholine) 20 mM, LDAO (lauryldimethylamine-N-oxide) 20 mM, LMPG (lyso-myristoylphosphatidylcholine) 0.1%, each used at a single concentration well above the individual species' CMC. Two mixtures were also tested: DCC (1% DDM+0.2% CHS [cholesterolhemisuccinate]+0.6% CHAPS [3-[(3-cholamidopropyl)dimethylammonio]-1-propanesulfonate]); DC (1% DDM+0.2% CHS). Samples contained in 2 ml tubes were mixed by rotation overnight at 4°C, followed by centrifugation at 53K (Optima TLX Ultracentrifuge, Beckman) at 4°C for 90 min. Next, supernatant aliquots of 1 µl were subject to Western blot analysis with anti-His_6_-tag antibody (Abcam).

### Receptor binding assays

*E. coli* intact cells or their membranes (M) expressing CRFRs were incubated with increasing concentrations of CRF family ligands in triplicate, diluted in binding buffer (50 mM Hepes pH 7.5, 0.1% BSA, 1 mM EDTA), and either [^125^I-DTyr^0^]astressin, [^125^I-Tyr^0^,Glu^1^,Nle^17^]-sauvagine or [^125^I-Tyr^0^,Glu^1^]PD-sauvagine (in binding buffer plus 0.02% Triton X-100) in GV or FB 96-well plates (Millipore) pre-wetted with 0.1% polyethyleneimine and washed with buffer, in a final volume of 120 µl [55]. The incubation was performed at room temp for 90–120 min, at which time the plates were aspirated and each well was washed with 2×100 µl assay buffer, dried and counted in a γ-counter. Each assay included at least triplicate wells for each concentration, and the assays were repeated at least twice (except where indicated). The astressin saturation data, which were used to calculate the K_d_ for labeled astressin from saturation experiments, were obtained by incubating the receptors with increasing concentrations of radioligand; the non-specific binding was determined as cpm remaining in the presence of 300–1000 nM unlabeled ligand. Data for labeled PD-sauvagine show that the system does not saturate at nanomolar peptide concentrations (Figure S3); thus, the K_d_ for the ^125^I-labeled PD-sauvagine was not determined from saturation experiments.

The saturation and competitive binding experiments are analyzed by non-linear regression with the GraphPad Prism program (www.graphpad.com) assuming binding to a single site. Specifically, for saturation binding experiments, binding is determined as a function of increasing concentrations of radioligand. The equation used is y = {(B_max_)^.^[radioligand]}/{Kd+[radioligand]}, where y = the specific binding (i.e., total binding-non-specific binding). Competitive binding experiments use a constant concentration of radioligand together with increasing concentrations of competitor. The equation used is y = B_max_+{(B_max_-B_min_)/(1+10^x-Log(EC^_50_^)^)}, where y = cpm bound, B_max_ = cpm bound in absence of competitor, B_min_ = cpm bound at highest concentration of competitor, x = concentration of competitor and EC_50_ = effective concentration for 50% displacement. The inhibitory binding constant, K_i_ is calculated from the equation K_i_ = EC_50_/(1+[radioligand]/K_d_) where K_d_ is the affinity constant of the radioligand. Because [radioligand] is <K_d_, the K_i_ = EC_50_. For multiple determinations, Log (EC_50_) values were averaged to calculate the values in Table 1. In the competitive binding experiments, the specific activity of the radioligand was 2100Ci/mmol and the concentration of either labeled astressin or labeled PD-sauvagine was 0.4 nM–0.8 nM. In Table 1, the inhibition constant (K_i_) values are shown together with the standard error and ranges, calculated assuming log normal distribution.

## Discussion

In the *E. coli* host, several factors may affect both the yield of recombinant membrane protein and its distribution between membrane and inclusion bodies such as transcriptional promoter, translational initiation efficiency, codon usage, mRNA secondary structure and stability, protein translocation efficiency, protein stability, and its level of toxicity to the bacterial host. Important experimental variables consist of the choice of protein fusion partner, bacterial strain, growth medium, concentration of inducer, cell density at induction time point, expression time and temperature, and co-expression of chaperon proteins. A full evaluation of all variables is challenging because it would require a combinatorial optimization. In practice, structural biology of GPCRs requires a sufficient amount of the purified protein, typically on the order of several milligrams. The results relative to the expression of the mCRFR2β protein product represent a promising step toward the expression and purification of this GPCR. In contrast to mCRFR2β, the expression level reached by hCRFR1α might require further optimization. The lower expression level of hCRFR1α, observed across all experimental tested conditions, which included the use of strain Rosetta2(DE3), which supplements tRNAs for seven *E. coli* rare codons, cannot be ascribed to a higher occurrence or to a less favorable distribution of rare codons in the hCRFR1α amino acid sequence (data not shown). The fact that the two receptors' sequences diverge extensively at the N-terminal region corresponding to the first 20 aa of the mature hCRFR1α and to the first 35 aa of the mature mCRFR2β, but are highly similar (72% identical and 85% functionally conserved) as far as the rest of the amino acid sequence, would point to a negative effect exerted by the hCRFR1α N-terminal region, either at the RNA or at the protein level. However, the expression of CRFRs deletion variants showed that, following the deletion of the entire ECD-1, a lower level of hCRFR1α is still observed in comparison to mCRFR2β. This indicates that more subtle differences residing in the transmembrane or loop regions of the receptors sequences may be at the basis of the effect.

Interestingly, the crystal structure of the transmembrane domain (aa 104–373) of hCRFR1α [46] has been recently reported and in this breakthrough study the receptors have been produced in insect cells. In this respect, our results relative to the expression of CRFRs in bacterial membranes may allow stable isotope labeling of receptor molecules for NMR studies.

A common strategy to maximize the expression level of recombinant membrane-integrated GPCRs in the *E. coli* host consists of introducing a bacterial protein, such as maltose binding protein (MBP), including its SP, at the GPCR N-terminus. In some cases a protein domain, such as thioredoxin (TrxA), has been added at the C-terminus as well [29]. Class B GPCRs differ from the majority of the receptors because they include the large N-terminal ECD-1 [51]. In this work we have assumed that the presence of an endogenous folded domain at the N-terminus might eliminate the need of introducing an additional protein at the GPCR N-terminus. In addition, we reasoned that the presence of an N-terminal fusion protein might interfere with the ECD-1 folding or hinder the binding of CRFRs ligands. However it remains to be explored whether the addition of a fusion protein at the N-terminus (with or without a SP sequence) and/or at the C-terminus, as well as the use of alternative expression parameters, may increase the level of functional receptors expressed in *E. coli*. In this respect, the membrane-integrating domain Mistic has been previously utilized [56], while cell-free production systems represent an interesting alternative approach for the expression of CRFRs [57], [58].

The great majority of GPCR sequences do not contain an N-terminal SP. It follows that, in the absence of a SP, the N-terminal tail (N-tail) region of the membrane protein has to be translocated across the eukaryotic endoplasmic reticulum in a post-translational manner. However endogenous SP sequences are usually present in that minority GPCRs, such as class B1 CRFRs, which are characterized by a long N-tail [51]. Since the presence of the SP in eukaryotic cells ensures that the N-tail is translocated co-translationally, it has been speculated that long N-tail GPCRs may contain a SP because such long N-tails would not translocate effectively in a post-translational manner, possibly because they may contain a rapidly folding domain [59], [60]. Deletion experiments on human endothelin B receptor [59], cannabinoid receptor 1 [61], and VPAC1 receptor [62] have indicated that in these cases SP is either required for, or facilitates, the N-tail translocation across the endoplasmic reticulum, while in the case of rat CRFR1α the SP has been shown to promote the receptor expression level [63]. In the case of recombinant long N-tail GPCRs produced in *E. coli*, the rationale for adding a bacterial SP to recombinant long N-tail GPCR appears threefold. First, in contrast to eukaryotic organisms, inner membrane proteins with extracellular N-tails and without SP are uncommon in *E. coli*. Second, in absence of SP and in conditions of protein overexpression, a rapid cytoplasmic folding of the N-tail region might limit the translocation of the N-tail sequence. Finally, in absence of a timely translocation the N-tail region might be more vulnerable to the action of cytoplasmic proteases. These factors prompted us to incorporate a SP in the expression vector for recombinant CRFRs. In spite of these hypotheses, our results demonstrate that, in the tested conditions, PelB has a negative impact on the expression level of full-length membrane-integrated products for both receptors. Similarly, the addition of DbsA SP significantly reduces the full-length receptors' yield. However the results relative to the N-terminally truncated receptors, which are lacking the large ECD-1, clearly show that, at least in the case of hCRFRα, PelB increases the recombinant protein expression level. In conclusion, the presence of PelB SP limits the yield of full-length receptors, but enhances the expression level of ECD-1 deleted hCRFRα receptor. When evaluating the effect of a SP on the expression level of GPCRs produced in *E. coli*, it is important to point out that the effects on protein translocation may be confounded by other features of the sequence, both at RNA and at protein level. Our results point to the importance of SPs as one of the critical parameters for the optimization of the expression of GPCR, or other recombinant membrane proteins whose N-terminus is similarly located in the periplasmic space. Finally, it remains to be seen whether GPCRs produced in the *E. coli* host with or without PelB SP differ in their functional binding characteristics.

FC-14 detergent has been shown to be quite efficient in the membrane extraction of various GPCRs proteins. Screenings recently conducted on CCR5-CCR3-CXCR4-CX3CR1 chemokine receptors and the human olfactory receptor 17-4 have identified FC-14 as the detergent of choice for their solubilization from *E. coli* [37] and human cell membranes respectively [64]. In addition, this detergent is perfectly compatible with NMR studies. Still, detergents such as FC-12 and FC-14 may be relatively harsh and not optimal for crystallization. In such instances they may be either used in combination with milder detergent species, or exchanged with other types of detergents following the membrane extraction step.

The cloned CRFRs expressed in mammalian cells bind with nanomolar affinities both agonists such as sauvagine and CRF, as well as antagonists such as astressin [14], [55]. Astressin binds predominantly to the ECD-1s of the CRFRs, with some contribution to the binding from their juxtamembrane regions; sauvagine's binding is determined mainly by the juxtamembrane regions as shown by the fact that it does not bind to the isolated ECD-1s [7], [65]. Further, the CRFRs' coupling to G-proteins in mammalian cells is important for the high affinity binding of agonists, such as sauvagine and CRF, but not for the binding of antagonists such as astressin or the agonist Ucn1. In mammalian cells, most agonists show K_i_'s for binding to CRFRs that are higher when assayed by competitive displacement of labeled astressin compared to their K_i_'s when assayed using labeled sauvagine. The observation that the K_i_'s for competitive displacement of astressin bound to the *E. coli* membranes are greater than those for the mammalian receptors (i.e., that the apparent affinities are lower for receptors expressed in *E. coli*) may be a result of the fact that *E. coli* membranes do not contain G-proteins, as well as the fact that the radioligand used for displacement is an antagonist, namely astressin. Furthermore, CRFRs express putative N-glycosylation sites in their ECD-1s, whereas the receptors in *E. coli* are not glycosylated. The absence of glycosylation may also contribute to the difference in ligand affinity and specificity of the receptors in *E. coli* compared to that observed with receptors in mammalian cells. In addition, ligand affinities may be modulated by the state of oligomerization of the mammalian CRFRs.

Although characterization of binding determinants of PD-sauvagine in mammalian cells have not yet been published, the sequence of PD-sauvagine is highly homologous to that of sauvagine so that its binding determinants may be assumed to be similar. An important question when considering the expression of GPCRs in bacteria is whether the conformations of the transmembrane domains of those receptors are comparable to those of the native mammalian receptors. The availability of the new radioligand, ^125^I-labeled PD-sauvagine, which binds to the receptors in the *E. coli* membranes, has provided a tool to consider the question. The small molecule antagonist antalarmin binds to a site defined by residues in transmembrane domains 3 and 5 of CRFR1 in mammalian cells [66]. We have found that antalarmin competitively displaces labeled PD-sauvagine bound to a small percentage of the hCRFR1α expressed in *E. coli* membranes. This observation provides support for the conclusion that there is an antalarmin-binding site in the transmembrane domains 3 and 5 of hCRFR1α in the *E. coli* membranes and that therefore, there is a subset of the receptors that do have those correctly folded transmembrane domains. It is possible that the absence of G-proteins in *E. coli* results in a smaller percentage of correctly folded transmembrane domains. Recent crystallographic studies comparing the structure of a receptor bound to an inverse agonist with that of the un-liganded receptor have suggested that in the absence of ligand, the predominant form of the receptor is an inactive one and that only a small fraction of the receptors are in an active conformation; the active conformation is then stabilized by binding to the ligand followed by association with G-proteins [67].

In conclusion, the data presented in this manuscript showing similar specificity and selectivity for the receptors produced in *E. coli* support the usefulness of these proteins for further structural studies.

## Supporting Information

Figure S1 **Influence of BL21(DE3) vs. Rosetta2(DE3) on the expression of CRFRs in TB medium.** Expression of PelB-hCRFR1α (R1) and PelB-mCRFR2β (R2) was carried out in TB medium either in BL21(DE3) or in Rosetta2(DE3) strain. Equivalent volumes of a tenfold dilution of the bacterial inclusion bodies (IB) and membrane (M) fractions were analyzed by Western blot with His_6_-tag antibody. The results of two independent protein expressions in Rosetta2(DE3) strain (denoted I and II), are shown.
(TIF)

Figure S2 **Expression of recombinant CRFRs with DsbA signal peptide.** Comparative analysis of DsbA-hCRFR1α (R1) and DsbA-mCRFR2β (R2) expression vectors. Expressions were carried out in TB medium and Rosetta2(DE3) strain. Equivalent volumes of a tenfold dilution of the bacterial inclusion bodies (IB) and membrane (M) fractions were analyzed by Western blot with His_6_-tag antibody.
(TIF)

Figure S3 **Saturation binding of labeled PD-sauvagine.** Binding of increasing concentrations of labeled PD-sauvagine bound to (A) hCRFR1α or (B) mCRFR2β expressed in *E. coli* membranes. (◼) total binding; (♦) non-specific binding; (▲) specific binding.
(TIF)

Figure S4 **List of oligonucleotides.**
(PDF)
