# Supplementary figures and images for: Expression and Functional Characterization of Membrane-Integrated Mammalian Corticotropin Releasing Factor Receptors 1 and 2 in Escherichia coli
Source: PLoS One. 2014 Jan 17;9(1):e84013. doi: 10.1371/journal.pone.0084013 (PMC3894963; doi:10.1371/journal.pone.0084013)

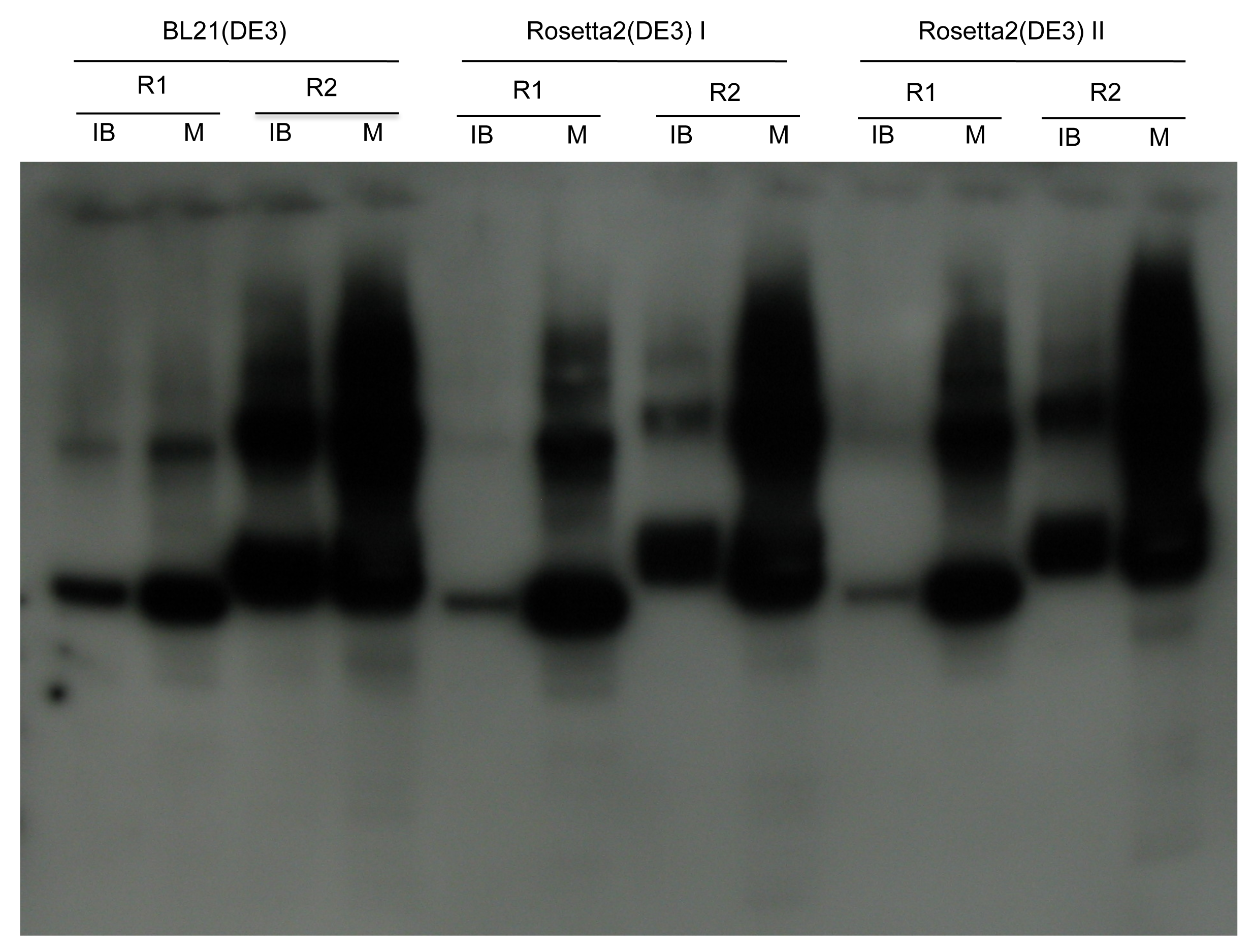

Supplement: Figure S1 — Influence of BL21(DE3) vs. Rosetta2(DE3) on the expression of CRFRs in TB medium. Expression of PelB-hCRFR1α (R1) and PelB-mCRFR2β (R2) was carried out in TB medium either in BL21(DE3) or in Rosetta2(DE3) strain. Equivalent volumes of a tenfold dilution of the bacterial inclusion bodies (IB) and membrane (M) fractions were analyzed by Western blot with His6-tag antibody. The results of two independent protein expressions in Rosetta2(DE3) strain (denoted I and II), are shown. (TIF) [file pone.0084013.s001.tif]

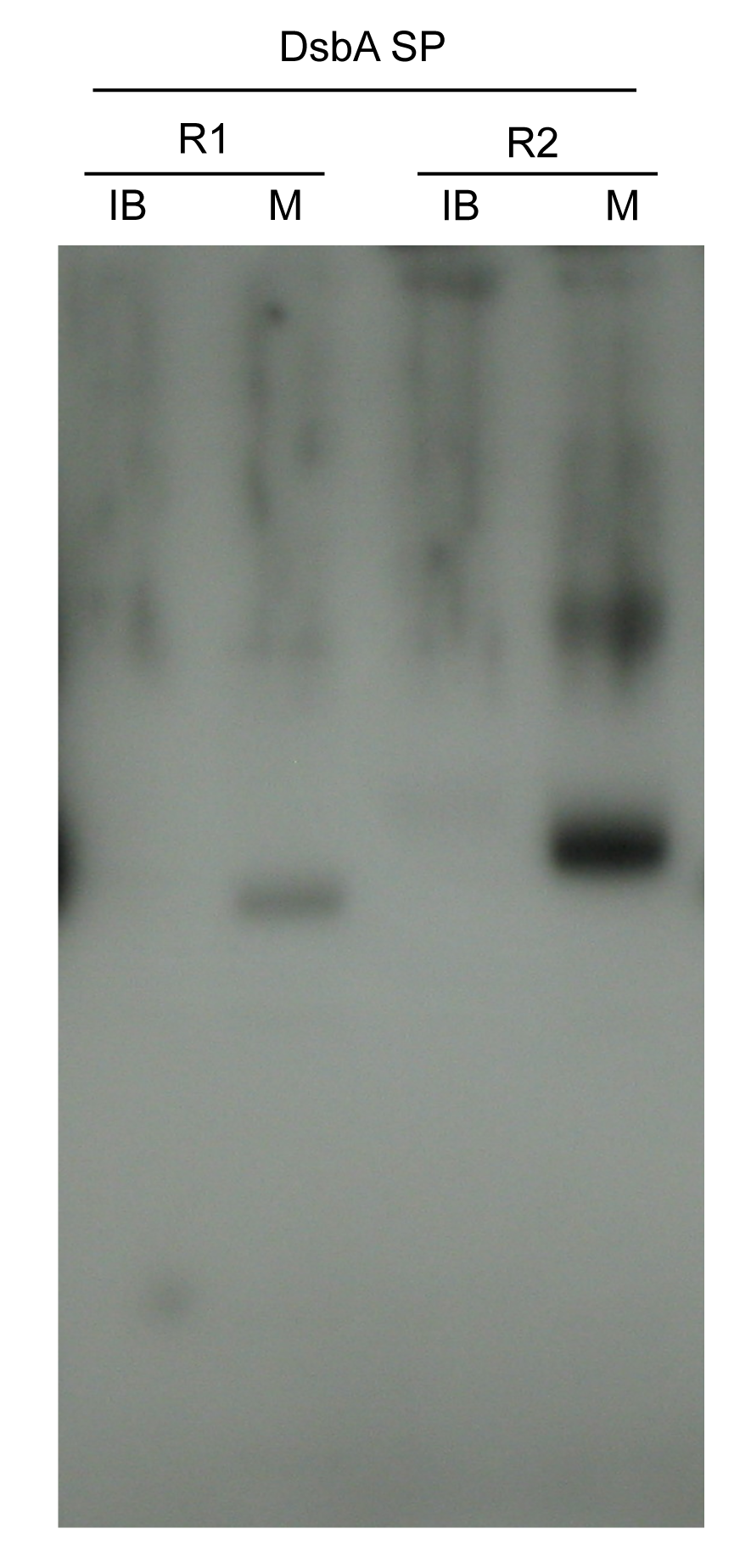

Supplement: Figure S2 — Expression of recombinant CRFRs with DsbA signal peptide. Comparative analysis of DsbA-hCRFR1α (R1) and DsbA-mCRFR2β (R2) expression vectors. Expressions were carried out in TB medium and Rosetta2(DE3) strain. Equivalent volumes of a tenfold dilution of the bacterial inclusion bodies (IB) and membrane (M) fractions were analyzed by Western blot with His6-tag antibody. (TIF) [file pone.0084013.s002.tif]

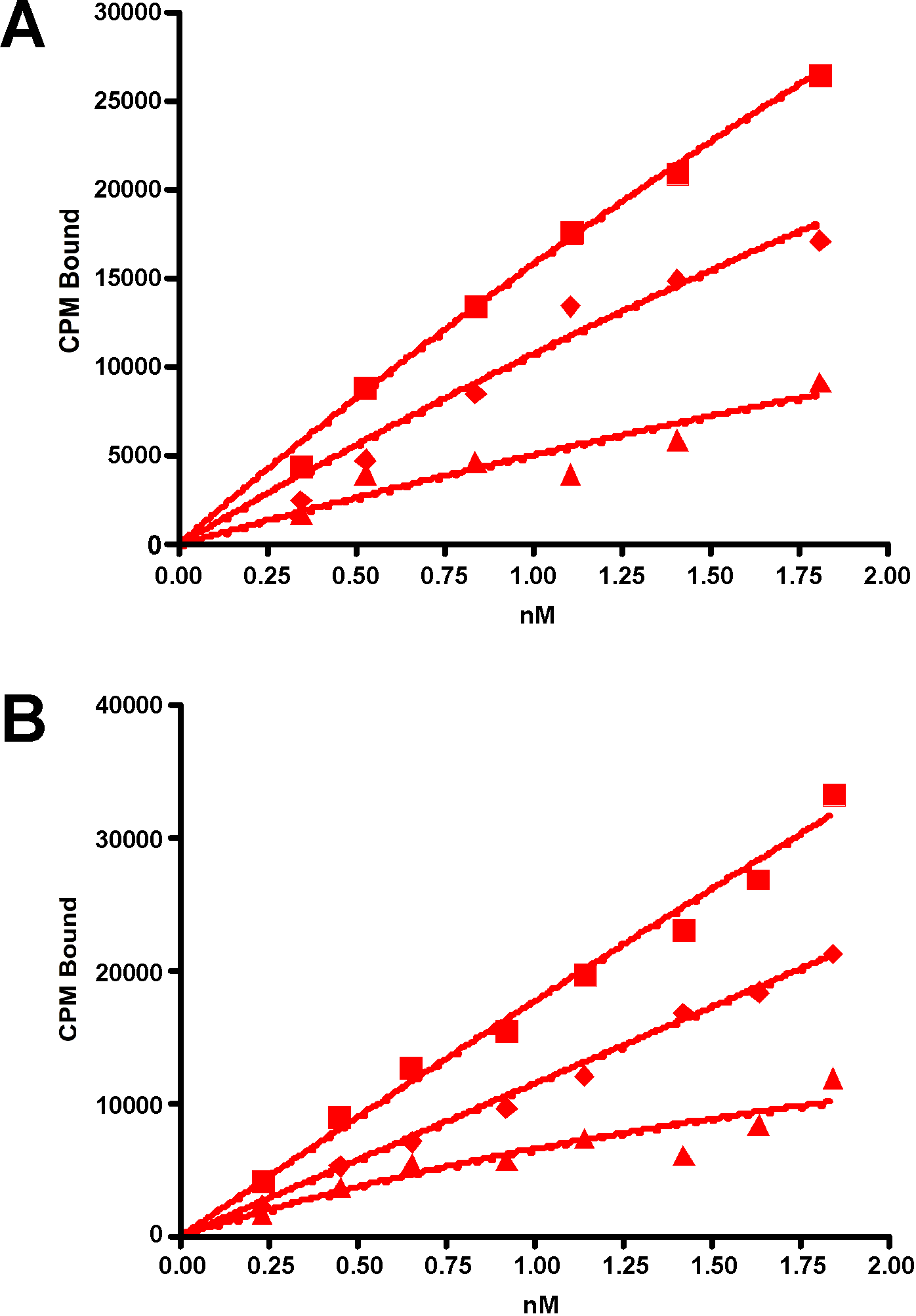

Supplement: Figure S3 — Saturation binding of labeled PD-sauvagine. Binding of increasing concentrations of labeled PD-sauvagine bound to (A) hCRFR1α or (B) mCRFR2β expressed in E. coli membranes. (◼) total binding; (♦) non-specific binding; (▲) specific binding. (TIF) [file pone.0084013.s003.tif]
