# Supplementary material for: Expression and Functional Characterization of Membrane-Integrated Mammalian Corticotropin Releasing Factor Receptors 1 and 2 in Escherichia coli
Source: PLoS One. 2014 Jan 17;9(1):e84013. doi: 10.1371/journal.pone.0084013 (PMC3894963; doi:10.1371/journal.pone.0084013)
Supplement: Figure S4 — List of oligonucleotides. (PDF) [file pone.0084013.s004.pdf]

Figure S4. Oligonucleotide sequences

C4 GCCCACTAGTCTCATTGCCCTCGTGCACTT,

C5 GCCCACTAGTCACCAAATCACCAGCTTCCT,

C6 GCCCACTAGTCACAATCTGGGACAGGTCGT,

C7 GCCCACTAGTCACAGCAGCTGTCTGCTTGA,

C8 ATTTGAGCTCGTGGCCCAACCAGGCCAGGC,

C11 TATTCCATGGTGGCCCAACCAGGCCAGG,

C12 CGCCGAATTCACAGCAGCTGTCTGCTTG,

C13 TATTCCATGGCCCTCCAGGACCAGCACT,

C14 CGCCGAATTCACTGCTGTGGACTGCTTG,

C27 TATGAAAAAGATTTGGCTGGCGCTGGCTGGTTTAGTTTTAGCGTTTAGCGCATCGGC,

C28 CATGGCCGATGCGCTAAACGCTAAAACTAAACCAGCCAGCGCCAGCCAAATCTTTTTCA,

C29 TATTCATATGGCCCTCCAGGACCAGCAC,

C30 TATTCATATGGTGGCCCAACCAGGCCAG,

C31 GCCCCATATGAAAAAAAGCAAGGTGCACTA,

C32 GCCCCATATGAGAAAGTATGACCTGCATTA,

C33 GCGACCATGGAGAAAAAAAGCAAGGTGCACTA,

C34 CCCGCCATGGAGAGAAAGTATGACCTGCATTA.
